# P051. Olfactory migrainous hallucinations: a typical aura manifestation?

**DOI:** 10.1186/1129-2377-16-S1-A80

**Published:** 2015-09-28

**Authors:** Matteo Bellamio, Federico Mainardi, Giulia Toldo, Giorgio Zanchin, Ferdinando Maggioni

**Affiliations:** Headache Centre, Department of Neurosciences, University of Padua, Padua, Italy; Headache Centre, Neurological Division, SS Giovanni e Paolo Hospital, Venice, Italy

## Introduction

Although olfactory hallucination or phantosmia could occur in several neurological and non-neurological conditions, olfactory migrainous hallucination (OMH) is a rare and probably underestimated phenomenon, involving about 0.1% of migraine patients[1], and not considered among the migrainous aura manifestations according to the International Classification of Headache Disorders, 3^th^ edition (ICHD-III beta version)[2]. Very few clinical studies on the topic have been published[3]; therefore, the clinical characterization of OMH is still lacking.

## Materials and methods

We report the clinical features of OMH prospectively collected by a detailed and structured anamnesis obtained in 5 patients who spontaneously referred the presence of OMH associated to their headache attacks. Patients were subsequently followed with a diary for at least a year. Moreover, the efficacy of the prophylactic therapy, if suggested, has been recorded.

## Results

Five patients (4 females, 1 male) presented with a history of migraine without aura (MO) (n=4) and with aura (MA) (n=1) associated with OHM. Mean age at the first evaluation and at headache onset was respectively 42.2 years (range 25-51) and 17.0 years (range 5-28), while OMH appeared at a mean age of 34.6 years (range 5-54). In 4 cases, a concomitant primary headache was diagnosed (MA, n=2; episodic tension-type headache, n=1; primary stabbing headache, n=1). Physical and neurological examinations, laboratory analyses, neuroimaging and EEG resulted unremarkable. OMH presented with an average frequency of once every 3 attacks. Onset and resolution of phantosmia were sudden in 3 cases and gradual in the remaining 2, with a mean duration of 10 min. The painful phase followed the disappearance of OMH in all the cases. The type of the perceived smell was invariably constant in 9 patients, while one patient reported different phantosmia for every different attack.

## Conclusions

When properly asked, patients are able to describe in detail the features of their olfactory hallucination. Their characteristics fulfilled the ICHD-III beta criteria for the aura symptoms[2]: if these features should find confirmation in further prospective studies, OMH could be considered similarly to the typical aura manifestations and included among them in the MA diagnostic criteria in the appendix of the next ICHD.

Written informed consent to publication was obtained from the patient(s).
